# Analysis of deep venous thrombosis after Gynecological surgery: A clinical study of 498 cases

**DOI:** 10.12669/pjms.312.6608

**Published:** 2015

**Authors:** Lihua Zhang, Xiancui Liu, Yunxia Xue

**Affiliations:** 1 Lihua Zhang, Department of Gynecology, The Third Hospital of Jinan Shandong Province, 250132, China; 2 Xiancui Liu, Department of Gynecology, The Third Hospital of Jinan Shandong Province, 250132, China; 3 Yunxia Xue, Department of Gynecology, The Third Hospital of Jinan Shandong Province, 250132, China

**Keywords:** Risk factors, Deep venous thrombosis (DVT), Gynecological surgery

## Abstract

**Objectives:**

To find out the clinical characteristics and risk factors for deep venous thrombosis (DVT) after gynecological surgery.

**Methods:**

Four hundred and ninety-eight patients treated surgically in the department of gynecology of our hospital from July 2012 to May 2014 were reviewed retrospectively. The data including patient age, gender, medical history, hospital stay, anesthesia type, operation time, occupation type, operative or postoperative medicine, perioperative bleeding, postoperative activity time, mortality rate and so on, were collected.

**Results:**

Among 498 patients, 58 were included in the thrombosis group, 423 patients in the non-thrombosis group and 17 patients were excluded. The incidence of deep venous thrombosis was 11.6%. In 58 cases with deep venous thrombosis, 6 cases developed pulmonary embolism and two patients died, the mortality rate for pulmonary embolism is 33.3%. In multivariate analysis, age, malignant tumor, cardiovascular comorbidity and postoperative hemostatics dose are independent risk factors, physical labour and minimally invasive surgery are protective factors for DVT.

**Conclusion:**

The patients with elder age, malignant tumor, cardiovascular comorbidity or large postoperative hemostatics dose should be paid high attention to and the minimally invasive surgery are optimal treatment in preventing DVT.

## INTRODUCTION

Deep vein thrombosis (DVT) and its associated pulmonary embolism(PE) present with major problems in the field of surgery, which remains a significant cause of postoperative mortality and morbidity.^1^,^2^ Gynecological surgical patients have high risks for developing deep venous thrombosis because they experience hypercoagulable states, immobility and vascular injuries during the course of their surgeries. In a prospective clinical study of 141 cases treated using gynecological surgery, Liu and colleagues reported 22 cases suffered from DVT and the incidence was 15.6%.^3^ In addition, the incidence of DVT is relatively higher in patients with gynecological tumors, and the risk of perioperative deep venous thrombosis was reported with ranges from 19.6% to 38% in patients with gynecologic cancers versus 10-15% in benign gynecologic tumors.^4^ PE is the leading cause of post-operative death after surgery for gynecologic cancer and a third of cases with DVT may develop PE that carries a fatality rate of 10%.^5^ Subsequently, the fatal complication has been paid high attention in the gynecological department.

Although the perioperative low molecular weight heparin (LMWH) applying in patient undergoing gynecological surgery completely eliminated DVT and PE incidence,^1^ DVT still occur after the gynecological surgery. In addition, the clinical diagnosis of DVT is notoriously inaccurate, with only 50% of cases being detected on the basis of signs and symptoms.^6^ The missed diagnosis often occurred and the early and accurate diagnosis were challenging in clinical practice. Subsequently, it is critical for gynecological surgeons to learn the clinical characteristics of DVT and risk factors related to DVT after gynecological surgeries. However, up to now, few clinical studies have been published on the issues in English literatures.

Therefore, in the current study, we retrospectively reviewed 498 patients treated using gynecologic surgery between July 2012 and May 2014. The aim of the current study was to find out the clinical characteristics and risk factors of DVT after gynecologic surgery, to help surgeons better understand and prevent the fatal complication during perioperative periods.

## METHODS

Four hundred and ninety-eight patients treated surgically in the department of gynecology of our hospital from July 2012 to May 2014 were reviewed retrospectively. The data including patient age, gender, medical history, hospital stay, anesthesia type, operation time, occupation type, operative or postoperative medicine, perioperative bleeding, postoperative activity time, mortality rate and so on, were collected.

The diagnosis of DVT was determined as clinically suspected DVT or PE confirmed by imaging and requiring therapeutic anticoagulation or resulting in death. Patients who had a recent DVT diagnosed prior to surgery and those who developed arterial thrombosis were excluded from the current study^5^ which was approved by the ethics committee of our hospital.

Statistical analysis was performed using SPSS 19.0 (SPSS Inc., Chicago, IL, USA). Independent 2-sample t test was carried out to compare the difference of measurement data, and a chi-square test were used to compare the difference of enumeration data between two groups. Univariate and multivariate logistic regression analysis were carried out to find the correlation between variables and DVT, and the multivariate logistic regression analysis was used to determine the independent risk factors for DVT. A probability value of < 0.05 was considered to indicate statistical significance.

## RESULTS

Among 498 patients, 58 were diagnosed as DVT and included in the thrombosis group, 423 patients were included in the non-thrombosis group and 17 patients were excluded from the current study. The incidence of DVT was 11.6%. In 58 cases with DVT, 6 cases developed PE and two patients died. The mortality rate for PE is 33.3%.

Among 58 cases, 11 were uterine fibroids, 13 were uterine adenomyoma, 14 were ovarian tumors, 10 were cervical cancer and 10 were ectopic pregnancy. The average age was 49.5 years old, ranged from 29 to 66 years old. The DVT occurred in fibular veins, calf muscular veins or posterior tibial veins, among which 17 cases in left lower extremity, 23 in right lower extremity and 18 in both lower extremities.

In the current study, the comparison of the clinical characteristics between the two groups are listed in Table-I. There was significant difference in age, cardiovascular comorbidity, surgical mode, occupation type, number of malignant tumors, operation time, postoperative activity time and postoperative hemostatics dose between the two groups, but no significant difference in body mass index (BMI) (Table-I). In addition, we found the age, occupation type, cardiovascular comorbidity, operation methods, malignant tumor and postoperative hemostatics dose were filtered as correlated factors for the occurrence of DVT in α<0.2 level according to univariate logistic analysis. In multivariate analysis, age, malignant tumor, cardiovascular comorbidity and postoperative hemostatics dose were independent risk factors, physical labour and minimally invasive surgery were protective factors for DVT (Table-II).

**Table-I:** The correlated factors of deep venous thrombosis after gynecological surgery.

Factors	Thrombosis n=58	Non-thrombosis n=423	P value
Age	53.8±4.9	42.3±3.7	0.023
BMI	24±3.1	26±4.5	0.21
Cardiovascular comorbidity (n/ %)	15(25.9%)	47(11.1%)	0.002
Occupation (physical/mental worker)	12/46	176/247	0.002
Surgical mode (laparotomy/Laparoscopic surgery or others, n/ %)	23/35(39.7.1%)	112/311(26.5%)	0.04
Malignancy (n, %)	25(43.1%)	81(19.1%)	0.0004
Postoperative activity time (hour)	61±3.8	49±4.5	0.01
Operation time (hour)	3.5±0.9	2.9±0.8	0.04
Postoperative hemostatics dose (U)	0.8±0.3	0.4±0.1	0.03

**Table-II:** The independent risk factors associated with deep venous thrombosis.

Factors	P value	OR (95% CI)
Age (≥45 years old)	0.04	2.5(0.9-13.1)
Occupation(physical/mental worker)	0.02	0.37(0.1-1.7)
Malignant tumor (n, %)	0.01	3.20(1.2-5.9)
Cardiovascular comorbidity (n/ %)	0.02	2.80(1.0-6.8)
Postoperative hemostatics dose	0.03	2.72(0.9-4.7)
Surgical mode (laparotomy/minimally invasive surgery, n/ %)	0.04	1.29(0.7-5.1)

## DISCUSSION

It is reported in the United States that two million people suffered from DVT each year and 600000 progresses to PE, in which 200000 were fatal.^7^ Venous thromboembolism is a major cause of morbidity and mortality in the world. In the current study, we performed a retrospective analysis of patients receiving gynecologic surgery, to determine the risk factors of DVT after surgery, to the best of our knowledge, few studies have been published on the issues.

Many studies advocated that DVT is related closely to injury of vessel wall, slowing of blood flow and blood hypercoagulation.^2^ Gynecologic surgery leads to a high risk of DVT, which may be attributed to many factors. The walls of pelvic veins are thin and the veins of rectum, bladder and the reproductive system are interlinked, resulting in the pelvic venous congestion and blood flow slowing. In addition, the anaesthesia during operation may cause venous distension and the long bed rest period after surgery may affect adversely the hemodynamics of the patients, aggravating the slowing of blood flow. The trauma resulted from operation also cause blood hypercoagulation negatively, and subsequently gynecologic surgery lead to the formation of DVT.^2^ The abovementioned reasons for the formation of DVT after gynecologic surgery has been confirmed by many authors.^2^,^4^,^5^

In the current study, the incidence of DVT after gynecologic surgery was 11.6%, lower than that reported by Liu, but close to that reported by Santoso.^3^,^4^ The relatively lower incidence may be attributed to the use of perioperative low molecular weight heparin (LMWH) in some patients. However, the mortality rate for PE in patients after gynecological surgery is as high as 33.3% in the current study. We found in some PE patients, the clinical symptoms are not so severe that the diagnosis of PE was neglected, which may be an explanation for the relatively high mortality rate of PE in the current study. However, in the two patients who died, PE occurred so suddenly that the rescues efforts were not successful. As a result, we suggest the fatal complication and its risk factors should be paid high attention to in patients receiving gynecological surgery, and detailed physical examination including imageological examination is critical.

Moreover, we found the elder age, malignant tumor, cardiovascular comorbidity, laparotomy operation and postoperative hemostatics dose were independent risk factors for the formation of DVT. In our opinion, these factors may be closely associated with the blood hyperagulation status, longer operation time, longer bed rest, more blood loss and more severe surgical strike, which surely aggravate the risk of DVT. In addition, we found the physical labour is a protective factor for DVT. In literatures, Yang^8^, Li^9^ and Liu^10^ also focused on the issues and concluded the similar conclusion. Different from the above mentioned studies, we found the minimally invasive surgery is also a protective factor in preventing the occurrence of DVT. Gynecologic laparoscopy has been regarded as a low-risk procedure for postoperative DVT,^11^ which was associated with shorter operation time, less blood loss and minimal surgical strike. In the current study, the rate of DVT in patients receiving laparoscopy treatment was significantly lower. Consequently, we suggest that the minimally invasive surgery is a better option for patients in gynecology department.

As DVT is often asymptomatic and in many patients, clinical presentation only occurs after a fatal PE, the prevention applied in patients undergoing gynecological surgery using low molecular weight heparin (LMWH) is optimal and necessary.^1^,^12^ However, some authors have different viewpoints. In a study of 419 patients who underwent minimally invasive surgery for a gynecologic cancer, 67 received DVT prophylaxis and 352 didn’t, the rate of DVT in the 352 untreated patients was 0.57% and no DVT diagnosed in the 67 patients who received anticoagulant thromboprophylaxis, Bouchard-Fortier G and colleagues suggest, as the rate of DVT is low in patients undergoing minimally invasive surgery for a gynecologic malignancy despite no DVT prophylaxis, the benefits of routine use of prophylaxis are questionable.^13^ In our opinion, the study of Bouchard-Fortier G and colleagues demonstrated that the surgical method played an important role on the final clinical outcomes, which is consistent with our study. However, as the rate of DVT is low in patients receiving minimally invasive surgery,^11^ a larger sample may be needed to clarify the difference between the two groups in Bouchard-Fortier G’s study. In the current study, we found the postoperative hemostatics dose was an independent risk factor for DVT, indicating that the blood coagulation status is critical during the occurrence and development of DVT. At the same time, we found the two dead patients didn’t received DVT prophylaxis. Although the small sample can’t demonstrate a statistical significance, we suggest the prophylaxis of DVT should be carried out postoperatively.

In addition, the current study was carried out retrospectively instead of prospectively, a prospective study may be better in clarifying the related issues. Despite it, we concluded from the current study that age, malignant tumor, cardiovascular comorbidity and postoperative hemostatics dose were independent risk factors, physical labour and minimally invasive surgery were protective factors for DVT, which may help surgeons better understand and prevent the fatal complication during perioperative periods.

## References

[1] Cyrkowicz A (2002). Reduction in fatal pulmonary embolism and venous thrombosis by perioperative administration of low molecular weight heparin. Gynecological ward retrospective analysis. Eur J Obstet Gynecol Reprod Biol.

[2] Tang S (2013). Pulmonary embolism after a single-stage, combined anterior and posterior approach lumbar surgery. Pak J Med Sci.

[3] Liu Y, Zhang Z, Guo S, He W, Zhang X, Wang S (2006). The clinical study on the deep venous thrombosis after gynecological surgery. Gynecol J China.

[4] Santoso JT, Evans L, Lambrecht L, Wan J (2009). Deep venous thrombosis in gynecological oncology: incidence and clinical symptoms study. Eur J Obstet Gynecol Reprod Biol.

[5] Peedicayil A, Weaver A, Li X, Carey E, Cliby W, Mariani A (2011). Incidence and timing of venous thromboembolism after surgery for gynecological cancer. Gynecol Oncol.

[6] Brennand JE, Greer IA (1998). Thromboembolism in gynaecological surgery. Curr Obstetr Gynaecol.

[7] Gross PL, Weitz JI (2008). New anticoagulants for treatment of venous thromboembolism. Arterioscler Thromb Vasc Biol.

[8] Yang BL, Zhang ZY, Guo SL (2009). Clinical significance of preventive treatment of thrombosis for patients undergoing gynecological surgery with high risk factors. Zhonghua Fu Chan Ke Za Zhi.

[9] Li L (2014). The analysis of risk factors for deep venous embolism after gynecological surgery. Guangxi Medicine.

[10] Liu Y, Zhang Z, Guo S, He W, Zhang X, Wang S (2006). The clinical study of deep venous embolism in gynecological surgery. China Gynecol.

[11] Ageno W, Manfredi E, Dentali F, Silingardi M, Ghezzi F, Camporese G (2007). The incidence of venous thromboembolism following gynecologic laparoscopy: a multicenter, prospective cohort study. J Thromb Haemost.

[12] Harrington D (2013). Preventing and recognizing venous thromboembolism after obstetric and gynecologic surgery. Nurs Womens Health.

[13] Bouchard-Fortier G, Geerts WH, Covens A, Vicus D, Kupets R, Gien LT (2014). Is venous thromboprophylaxis necessary in patients undergoing minimally invasive surgery for a gynecologic malignancy?. Gynecol Oncol.

